# Mechanistic Insights into the Polymorphic Associations and Cross-Seeding of Aβ and hIAPP in the Presence of Histidine Tautomerism: An All-Atom Molecular Dynamic Study

**DOI:** 10.3390/ijms23041930

**Published:** 2022-02-09

**Authors:** Abbas Salimi, Sompriya Chatterjee, Jin Yong Lee

**Affiliations:** Department of Chemistry, Sungkyunkwan University, Suwon 16419, Korea; salimi@skku.edu (A.S.); sompriya.chatterjee@gmail.com (S.C.)

**Keywords:** Alzheimer’s disease (AD), cross-seeding, histidine tautomerism, type 2 diabetes (T2D)

## Abstract

Hundreds of millions of people around the world have been affected by Type 2 diabetes (T2D) which is a metabolic disorder. Clinical research has revealed T2D as a possible risk factor for Alzheimer’s disease (AD) development (and vice versa). Amyloid-β (Aβ) and human islet amyloid polypeptide are the main pathological species in AD and T2D, respectively. However, the mechanisms by which these two amyloidogenic peptides co-aggregate are largely uninvestigated. Herein, for the first time, we present the cross-seeding between Amylin1-37 and Aβ40 considering the particular effect of the histidine tautomerism at atomic resolution applying the all-atom molecular dynamics (MD) simulations for heterodimeric complexes. The results via random seed MD simulations indicated that the Aβ40(δδδ) isomer in cross-talking with Islet(ε) and Islet(δ) isomers could retain or increase the β-sheet content in its structure that may make it more prone to further aggregation and exhibit higher toxicity. The other tautomeric isomers which initially did not have a β-sheet structure in their monomeric forms did not show any generated β-sheet, except for one seed of the Islet(ε) and Aβ40(εεε) heterodimers complex that displayed a small amount of formed β-sheet. This computational research may provide a different point of view to examine all possible parameters that may contribute to the development of AD and T2D and provide a better understanding of the pathological link between these two severe diseases.

## 1. Introduction

**Figure 1:**
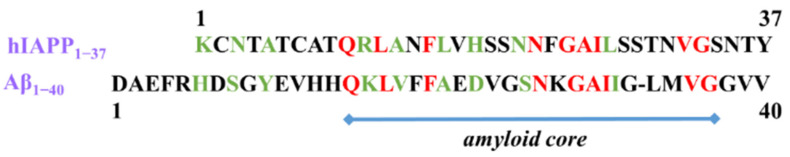
Sequence alignment of monomeric hIAPP1−37 and Aβ1−40. In both proteins, identical residual amino acids are marked in red and similar residues are highlighted in green.

In addition to the self-interactions mediating amyloid self-assembly, cross-seeding interactions between different amyloidogenic peptides may also play a vital role in the progression and transmission between diverse brain degenerative disorders [1]. Alzheimer’s disease (AD) and type II diabetes (T2D) are the two most common aging-related, chronic neurodegenerative disorders, both of which affect millions of people globally [2,3,4]. Several epidemiological and clinical studies have shown a strong association between AD and T2D based on their characteristic disorder symptoms [5]. AD patients exhibit a higher risk of developing T2D and vice versa [6]. Multiple mechanisms have been suggested to explore the AD–T2D link pathophysiologically, involving vascular inflammation, insulin resistance and deficiency, loss of cells connected with degenerative alterations, and glucose toxicity [5]. However, the exact clinical relationship between AD and T2D is still elusive [7]. At the molecular and protein level, it was recently shown that the AD–T2D association could arise from the cross-talk between the causative proteins connected with the two diseases, namely amyloid-beta (Aβ) and human islet amyloid polypeptide (hIAPP) [8]. Aβ protein, derived by N-terminal proteolytic cleavage of the transmembrane amyloid precursor peptide (APP), is the chief component of senile plaques observed in the brain of the AD patient [9]. In addition, hIAPP, a 37-residue hormone protein synthesized by the islet β-cells is believed to be a causative factor for T2D, in which hIAPP-mediated amyloid aggregates were noticed in over 95% of T2D patients [1]. The exact functions of hIAPP and Aβ proteins in the pathogenesis of T2D or AD remain largely undetermined, but the disorder symptoms arise to connect well with the existence of tiny accumulates of hIAPP or Aβ, often designated as soluble oligomers [10,11]. Monomeric hIAPP and Aβ play a significant role in oligomer formation. Toxic Aβ and hIAPP oligomeric aggregates attack cells in diverse routes to cause cell dysfunction and even death [12,13]. From a sequential or conformational point of view, both Aβ and hIAPP share sequence identity and similarity of 25 and 50%, respectively (Figure 1). Under the disordered state, they both share several organizational, toxicity, and kinetic properties during amyloid generation [14,15]. First, both Aβ and hIAPP are present in the blood vessels and cerebrospinal fluid (CSF) incomparable nanomolar concentrations, hence an in vivo cross-connection among them is possible, which form heterogeneous Aβ−hIAPP aggregates [1]. Second, Aβ and hIAPP can misfold and accumulate into structurally similar U-bend fibrils, consisting of two β-strands joined by a small turn. This unique β-rich conformer may provide a common conformational basis to initiate the cross-seeding associations between Aβ and hIAPP [7]. As the toxicity of amyloidogenic proteins including Aβ and hIAPP has been connected to its accumulation, it is likely that the Aβ−hIAPP accumulates may also possess some neurotoxicity. Regardless of the precise relation between AD and T2D, it is equally important to design some effective compounds to inhibit the formation of neurotoxic cross-talking amyloids [16]. Numerous in silico, in vivo, and in vitro studies have been conducted on Aβ and hIAPP cross-seed fibrillization. However, the exact correlation between the Aβ–hIAPP associations is still under investigation [17]. Wetzel and colleagues investigated the cross-talking connections between Aβ and hIAPP in vitro. They observed that the Aβ accumulates were good seeds for hIAPP fibrillization, but hIAPP seeds were almost inactive for Aβ accumulation, exhibiting distinct cross-interacting efficiencies based on the seeding fibrillar structure [18]. Furthermore, Andreetto et al. reported that hIAPP1−37 and its segments can connect with Aβ1−40 to form heterocomplexes that affect neurotoxic self-assembly by both peptides [14,19]. Seeding concentrations, solution conditions, sequence specificity, and agitation are the possible factors that may influence Aβ−hIAPP cross interaction [16]. Another aspect that may have an important effect is histidine tautomerization, which has been shown to play an important role in peptide aggregation. Neutral and protonated states of histidine are correlated with conformational features of diverse misfolded systems and can affect the fibrillization pathways in proteins, leading to proteopathies [20,21,22,23,24,25,26,27,28,29,30,31,32]. Surprisingly, there are few simulation studies on the cross-seeding of Aβ and hIAPP monomers to mimic the initial co-accumulation of both proteins [33,34]. In the current work, the influences of various histidine tautomeric forms on the cross-seeding of monomeric Aβ1−40 and hIAPP were investigated.

**Figure 2:**
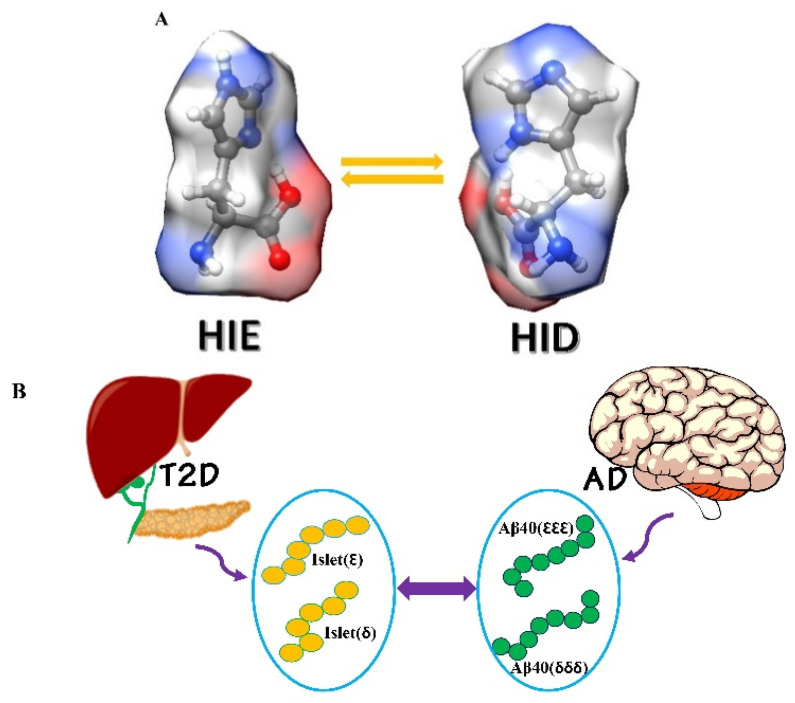
(**A**) Histidine in its diverse protonation states at neutral condition; and (**B**) possible combinations of Aβ1−40–hIAPP1−37 isomeric tautomers used in the current study.

Two neutral histidine tautomeric states [Nε2-H (ε) and Nδ1-H (δ); Figure 2A] can exist near pH 7, indicating the crucial biological phenomena occurring in a system [35,36]. Under neutral states, the population ratio of these two tautomers (ε/δ) is around 1:0.16 [37,38]. This ratio can be altered based on histidine position and side-chain (SC)–SC connection [39]. Interpreting the histidine tautomerism effect is essential for understanding how histidine plays organizationally and functionally important roles in polypeptides [35,36]. The structures were solvated using the TIP3P water model and neutralized using 1 Na^+^ ion before the MD run The structures were solvated using the TIP3P water model and neutralized using 1 Na^+^ ion before the MD run Numerous research groups have already proven the importance of the histidine tautomeric effects in some enzyme catalytic reactions, proton conductions, and protein bioactivity [40,41]. However, NMR spectroscopy is an effective technique for recognizing different histidine tautomers. In polypeptides, the situation becomes complicated: both the ^13^C and ^15^N chemical shifts of the histidine are very sensitive to its tautomeric states, the chemical environment of the aromatic rings, peptide backbone conformation, and hydrogen-bond lengths. In addition, the deprotonation and protonation of the histidine imidazole moieties occur on the picosecond time scale, quicker than NMR [42]. Hence, thorough experimental interpretation of tautomerism mediated cross-interaction of Aβ−hIAPP is difficult. Given the experimental limitations to obtain the atomic resolution of hybrid Aβ–hIAPP monomers due to the fast kinetics of amyloid accumulation in presence of the tautomerism, atomistic simulations provide critical insights into the structure and dynamics of hybrid Aβ–hIAPP under the histidine tautomeric effect [43]. Conventional molecular dynamics (MD) simulation is a powerful approach to gain a better understanding of the atomistic level details connected with amyloid cross-seeding [34]. Several labs have already performed classical all-atom explicit simulations to get atomic-resolution structures of hybrid Aβ-hIAPP assemblies [44,45]. Hansmann and co-workers explained that Aβ and hIAPP can connect through protein addition along the fibrillar axis [44]. Miller and colleagues noticed that Aβ and hIAPP oligomers can associate with each other to produce single- and double-layer heterocomplexes, with single-layer conformers being more convenient [45]. The monomeric Aβ1−40 contains three histidine residues located in the N-terminal domain of the protein at positions 6, 13, and 14, contributing to fibril stability at neutral pH [46]. On the other side, the hIAPP1−37 monomer comprises one histidine residue at the 18th position of the peptide sequence. This single amino acid plays a crucial role in enhancing H_2_O_2_ formation during hIAPP fibrillization [47]. As each histidine involves two tautomeric states (δ and ε), two isomeric forms exist in hIAPP1−37. For Aβ1−40, we used the highest toxicity δδδ and most common εεε isomer to investigate its interaction with hIAPP1−37 [20]. Considering Tau and Pi tautomeric forms of histidine in our work, Tau and Pi tautomers are denoted as ε and δ, respectively. Previous studies have shown that the protonation state of histidine modulates the Aβ and hIAPP protein’s accumulation, misfolding, and fibrillization properties [48,49]. Zweckstetter and co-workers reported histidine protonation induced Aβ and IAPP-GI association [50]. Additionally, regarding the unprotonated state, in our earlier studies, we already proved the effectiveness of the tautomeric state in neutral histidine residue in Aβ and hIAPP individually [31]. However, under neutral situations, the influence of the tautomerism condition in histidine amino acids during Aβ-hIAPP cross-interaction is largely unknown. The present work is the first research focused on the histidine tautomerism effect on cross-interaction between diverse misfolded peptides. Altogether, knowledge of conformational details of Aβ1−40–hIAPP1−37 complexes (Figure 2B) derived from this study provides a deeper understanding of the cross-sequence Aβ–hIAPP hetero-connections that may interpret a potential molecular link between AD and T2D and offers some clues to design strategies that will help to block the Aβ and hIAPP interaction.

## 2. Results and Discussion

### 2.1. Secondary Structure

**Figure 3:**
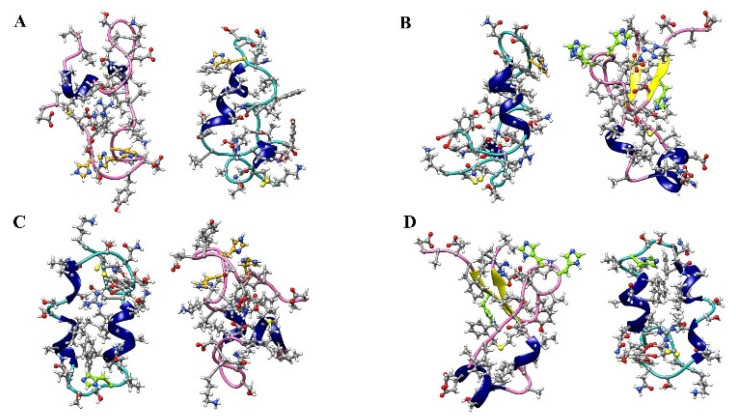
The initial structures of amylin-Aβ40 dimers were used for MD simulations. (**A**) Islet(ε)-Aβ40(εεε), (**B**) Islet(ε)-Aβ40(δδδ), (**C**) Islet(δ)-Aβ40(εεε) and (**D**) Islet(δ)-Aβ40(δδδ) conformations. Amylin, Aβ40, helix structures, HIE and HID residues are shown in light green, hot pink, navy blue, goldenrod, and chartreuse color, accordingly.

**Figure 4:**
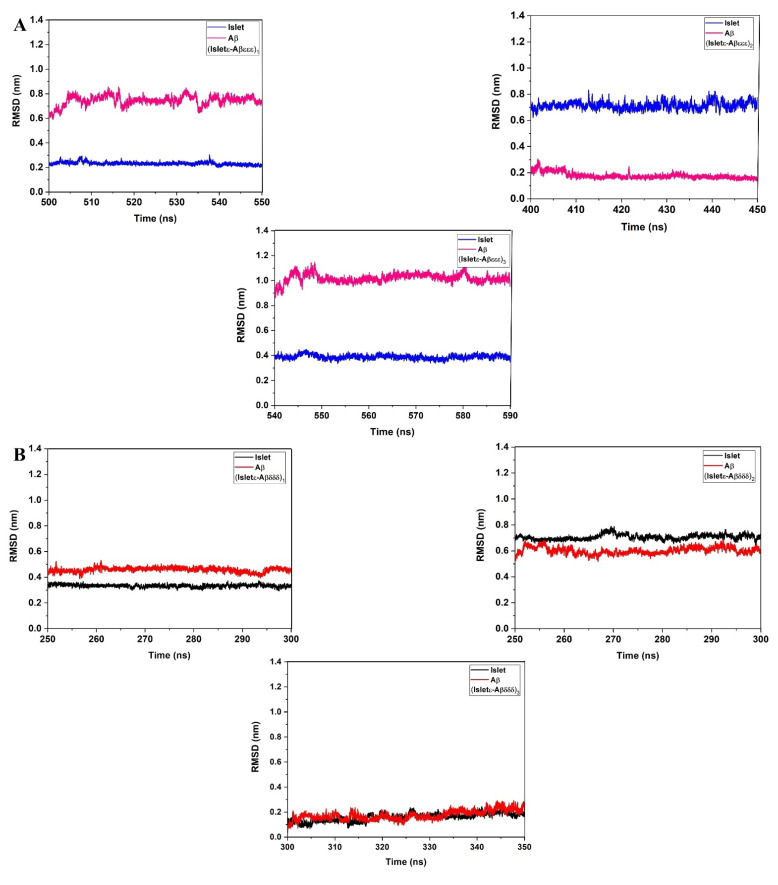

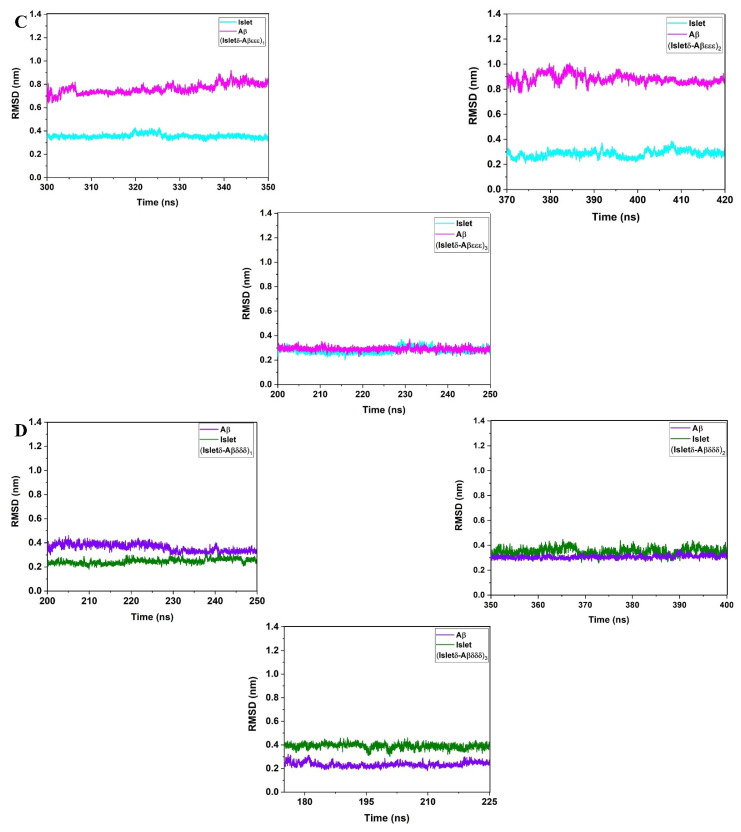
Root mean square deviation (RMSD), (**A**) Islet(ε)-Aβ40(εεε), (**B**) Islet(ε)-Aβ40(δδδ), (**C**) Islet(δ)-Aβ40(εεε), and (**D**) Islet(δ)-Aβ40(δδδ) cross-seeds.

**Figure 5:**
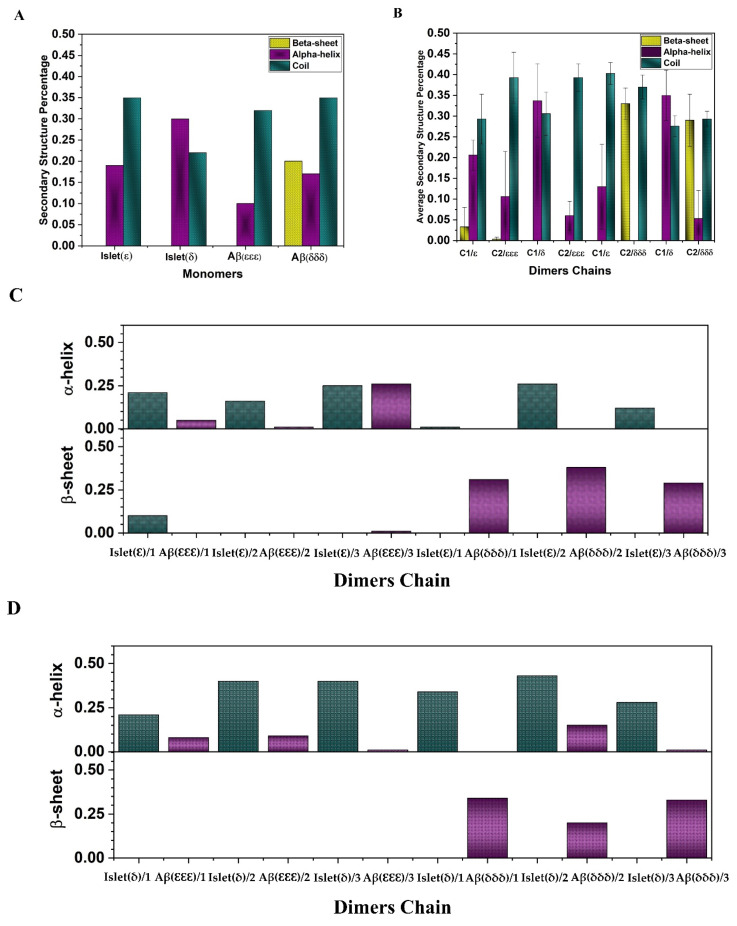
(**A**) The initial secondary structure percentage of amylin and Aβ40 monomers before MD simulations. (**B**) Average secondary structure of each chain considering the 3 seeds (amylin and Aβ40 are shown as C1 and C2, respectively). Error bars indicate the standard deviation. (**C**) α-helix and β-sheet content of each chain in dimer conformations for all seeds. (**D**) dimers formed by Islet(ε)-Aβ(εεε) and Islet(ε)-Aβ(δδδ) and bottom) dimers formed by Islet(δ)-Aβ(εεε) and Islet(δ)-Aβ(δδδ).

A computational study on amylin-Aβ42 mixture interaction has revealed the heterodimers as the most dominant species at the initial stages of assembly before converting to the heterohexamer [51]. The initial structures of amylin-Aβ40 dimers that were used for MD simulations in the current work are shown in Figure 3. After performing the MD, considering the (root mean square deviation) RMSD plots (Figure 4) for all three random seeds of the possible isomeric combinations the last 50 ns of the equilibrated part was taken for further structural analysis. The secondary structure analysis using the dictionary of secondary structures of proteins (DSSP) algorithm was applied on the equilibrated section of the trajectories. Before conducting the MD simulation, the β-sheet, α-helix, and coil content of the monomers were calculated and shown in Figure 5A. The β-sheet only can be observed in the Aβ(δδδ) monomer (~20%) and Islet(δ) has the highest α-helix content (~30%) while in the other monomers the coil structure is the dominant form.

Three random seeds have been used to perform the MD simulations for each dimer conformation. It would be informative to investigate the structural features of the dimers during cross-interaction. The average β-sheet, α-helix, and coil formation for each dimer chain (amylin and Aβ) were obtained and depicted in Figure 5B. Looking at the results, one can see that in Islet(ε)-Aβ(εεε) conformation a very small amount of β-sheet in average was generated in both chains (~0.03% for amylin) while initially there was no β-sheet at the monomeric form and Islet(ε) is still dominated by the coil structure. Additionally, comparing the Islet(ε) monomer with the Islet(ε) isomer in dimerization with Aβ(εεε) showed almost the same amount of α-helix (~20%) while the coil content decreased to around 29% due to the interaction. However the α-helix and coil content during cross interaction with Aβ(δδδ) showed the opposite trend with about 13% for the coil and 40% for the α-helix. The Aβ(εεε) isomer in the Islet(ε)-Aβ(εεε) dimer showed a very small amount of β-sheet (~0.003%) and slightly increase in coil (~39%) compare to the initial monomeric form, meanwhile the α-helix content remained the same. The Islet(δ)-Aβ(εεε) dimer did not show any sheet formation and the α-helix was predominant (~35%). The percentage of the coil and helix content of Islet(δ) in cross interaction with Aβ isomers displayed a small increase compared with the initial structure. Here, the α-helix of Aβ(εεε) slightly decreased to 6% and the coil content was about 39% as well. No sheet formation was observed for Aβ(εεε) in this combination. In Islet(ε)-Aβ(δδδ) cross interaction, the β-sheet content of Aβ(δδδ) reached 33% compared to the 20% at the monomeric structure while the coil amount (~37%) increased a bit as well and no α-helix was observed. In Islet(δ)-Aβ(δδδ) contact, the Aβ(δδδ) β-sheet percentage was around 29% while Islet(δ) had no β-sheet. Meanwhile, the coil and α-helix content of Aβ(δδδ) decreased to 29% and 5%, accordingly. In the case of Islet(δ), the coil and α-helix compared to the monomer moderately increased to about 27% and 35%, respectively.

To get detailed information on the secondary structure of each random seed and chain, the calculated β-sheet and α-helix content are shown in Figure 5C,D. As it was depicted in Figure 5A,C,D, the Islet(ε) only in one seed showed the 10% of β-sheet formation among all seeds for both isomeric forms of amylin compared with to no β-sheet before cross interactions. Meanwhile, the α-helix amount slightly increased within the two seeds. In Islet(δ), the two seeds exhibited an increasing trend for the α-helix compared with the monomeric isomer and reached 40%. However, in the first seed, it decreased to around 21%. Regarding the Aβ(εεε) isomer, only the third seed showed 1% of β-sheet formation during dimerization while the others remained without sheet content, the same as the monomeric form. In addition, in cross interaction between Aβ(δδδ) with Islet(δ) and Islet(ε), Aβ(δδδ) in all seeds except the second seed that the β-sheet content of Aβ(δδδ) maintained the same amount as the initial monomeric structure revealed the increased amount (highest ~38%). Results may indicate that the amylin affected the Aβ40 β-sheet formation in the case of Aβ(δδδ) more by enhancing the content (except one seed) and this isomer could maintain its initial sheet amount and even experience increasing content. However, only one seed in the amylin isomer showed small β-sheet formation so probably the Aβ40 isomer also shows more stability than that of the amylin due to lower flexibility in β-sheet regions [45,52]. The results may also indicate the possibility of enhancing the toxicity of Aβ40(δδδ) due to increasing the β-sheet content in the presence of islet isomers and risk of AD. The clearance rate of Aβ in the brain of AD patients is notably lower than normal people [53]. Due to the histidine tautomeric effect on the structural characteristics of the amyloid and amylin that lead to the Aβ40(δδδ) isomer with a high content of β-sheet as a remarkable characteristic, we could speculate that this isomer of Aβ40 may participate more to form the higher-order oligomers and fibrils than other isomers and may facilitate further aggregation and insoluble structures generation. The Aβ structures rich in β-sheet could act as neurotoxic agents. This high aggregation potency may affect and change the clearance and balance pathway.

### 2.2. Contact Maps

**Figure 6:**
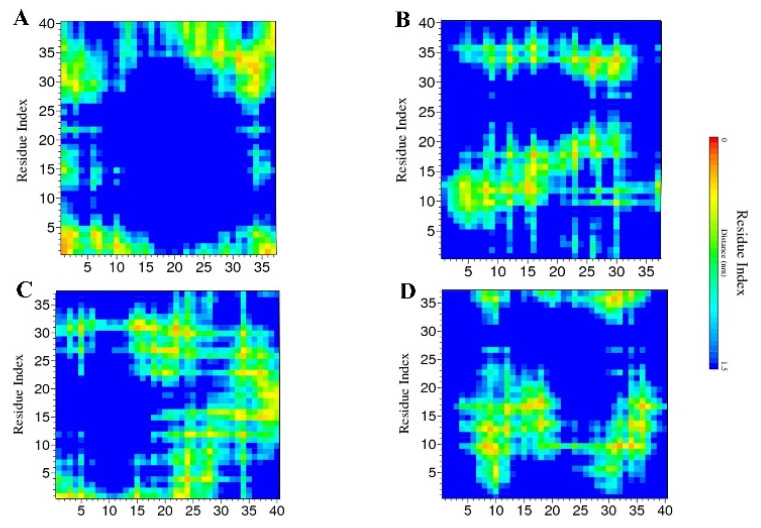
Contact maps of (**A**) Islet(ε)-Aβ40(εεε) 1st, (**B**) Islet(ε)-Aβ40(δδδ) 2nd, (**C**) Islet(δ)-Aβ40(εεε) 2nd and (**D**) Islet(δ)-Aβ40(δδδ) 1st conformations. X- and Y-axes display the residues indexes of the two chains.

To explore the major interacting sites between two chains in each monomer, due to the importance of β-sheet content during the aggregation progress and having the higher neurotoxicity, for each dimer the seed with the highest content of the β-sheet was selected to generate the contact maps. In the case of Islet(δ)-Aβ(εεε) conformation since no β-sheet was formed yet the seed with the highest α-helix amount considering the possible transition of α-helix toward β-sheet has been selected. So the contact maps have been made using GROMACS v.5.0 software for Islet(ε)-Aβ40(εεε) 1st, Islet(ε)-Aβ40(δδδ) 2nd, Islet(δ)-Aβ40(εεε) 2nd and Islet(δ)-Aβ40(δδδ) 1st conformations as shown in Figure 6. The distance between residues for a significant contact was considered around 0.6 nm.

In the first seed of Islet(ε)-Aβ40(εεε) some major contacts can be seen approximately between (1) residues K1-L12 of Islet(ε) with D1-R5 of Aβ40(εεε), (2) residues D1-R5 of amylin chain with S29-N35 of Aβ40(εεε), (3) S34-Y37 of Islet(ε) with D1-R5 residues of the 2nd chain, (4) N22-S28 of amylin with M35-V40 of Aβ40(εεε), (5) contacts between S29-T36 of the first chain with G29-V36 of the Aβ40(εεε), (6) N35-Y37 of Islet(ε) with V35-V40 residues of Aβ40(εεε) (Figure 6A). Regarding the histidine residues, the H6 and H14 residues in Aβ40(εεε) showed contact with the K1 residue of amylin (Figure 6A). In Islet(ε)-Aβ40(δδδ) 2nd seed, contacts between residues N3-T9 of Islet(ε) with G9-H13 of Aβ40(δδδ), Q10-H18 of Islet(ε) with V12-H13 of Aβ40(δδδ), F15-H18 of amylin chain with V12-V18 of Aβ40(δδδ), S20-T30 of Islet(ε) with residue F20 of the second chain and F23-V32 of amylin with the Aβ40(δδδ) chain are among the significant contacts (Figure 6B). Also, H18 in Islet(ε) had contacts with V12-F19 of Aβ40(δδδ) and H13 and H14 of Aβ40(δδδ) interacted with C2-H18 and N3-H18 residues of Islet(ε), respectively (Figure 6B).

Islet(ε)-Aβ40(δδδ) cross-interaction indicated some regions with significant interactions such as between the K1 residue of Islet(ε) with D1-D7 of Aβ40(δδδ), K1-T4 of Islet(ε) with D1-E3 of Aβ40(δδδ), N31-V32 of amylin with E3-D7 of the second chain, K1-T9 of the Islet(ε) chain with E22-K28 of the Aβ40(δδδ), L27-V32 of the Islet(ε) with Q15-G25 of Aβ40(δδδ), residues F15-L16 of Islet(ε) with K28-V40 of the second chain and N14-F23 of Islet(ε) chain with V36-V40 of Aβ40(δδδ) isomer (Figure 6C). In addition, H18 of Islet(ε) showed contacts with the G37-V40 region of Aβ40(δδδ) and H13 of Aβ40(δδδ) interacted with residues D1 and N31 of amylin isomer while H14 had some contacts with residues D1 and N31-V32 of the Islet(ε) as well (Figure 6C). The last contact map for the first seed of Islet(δ)-Aβ40(δδδ) revealed several closely contacted regions such as T4-H18 of Islet(δ) with D7-E12 of Aβ40(δδδ), A13-S20 of amylin chain with Q15-F19 of (δδδ) isomer, N36-Y37 of Islet(δ) with D7-Y10 of Aβ40(δδδ), N35-Y37 of the first chain with the region K28-L34 of Aβ40(δδδ) and A8-H18 of Islet(δ) with L34-V36 of Aβ40(δδδ) (Figure 6D). Specifically, H18 of Islet(δ) had contacts with Q15-F19 and L34-G37 of the second chain meanwhile residues H13 and H14 in Aβ40(δδδ) interacted with A13 and L16-L17 of the other chain (Figure 6D).

In all four maps, the residues in the N-terminal region of Aβ40 (D1-K16) exhibited some possible interactions with the amylin chain as it has been mentioned in another report in the case of Aβ42 and amylin cross-seeding as well that imply the importance of the N-terminals of both isomers during cross-interaction [45], which can be sometimes in favor of cross-interaction and other times they can be unfavorable [45]. Also, as it has been shown in another work, regarding the importance of residues L17-V24 and N27-A42 in Aβ42 and residues A8-H18 and N22-S28 for cross- and self-assembly [51], in the current maps we can see the interactions including these regions as well.

### 2.3. Cluster Analysis

**Figure 7:**
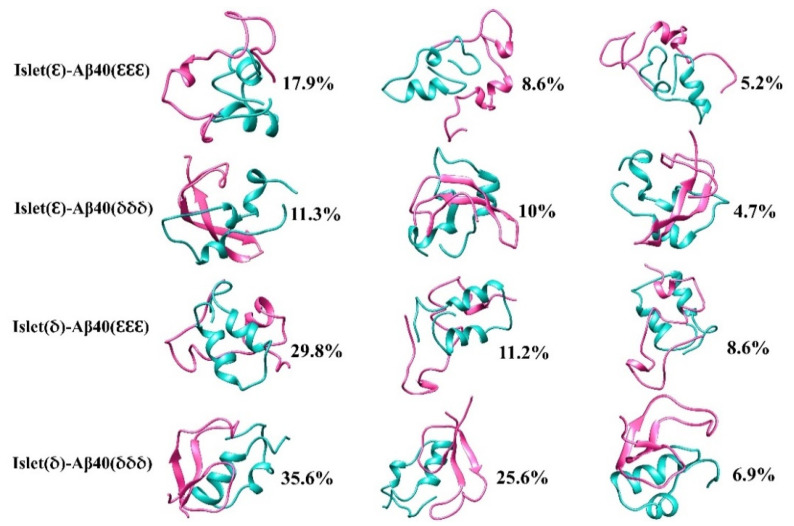
Representative structures and the percentage of distribution based on clustering of the selected seeds of each dimer conformation (Islet(ε)-Aβ40(εεε) 1st, Islet(ε)-Aβ40(δδδ) 2nd, Islet(δ)-Aβ40(εεε) 2nd and Islet(δ)-Aβ40(δδδ) 1st seed). The amylin and Aβ40 structures are shown in light green and hot pink, respectively.

To trace the conformational changes and dynamic properties of the structures [54] during the MD simulation the clustering analysis using the GROMOS [55] method implemented in GROMACS v.5.0 [56] software based on a 0.15 nm cut-off of backbone atoms was applied on the selected four seeds of dimers as mentioned in the prior section. The top three centroids from the most populated clusters of each selected dimer are shown in Figure 7. For instance, the analysis disclosed that the first two clusters in the Islet(δ)-Aβ40(δδδ) 1st seed accounted for around 62% of the conformation while in the Islet(δ)-Aβ40(εεε) 2nd seed this percentage was about 41%. In Islet(ε)-Aβ40(εεε) 1st, and Islet(ε)-Aβ40(δδδ) 2nd seeds the highest clusters were around 17.9% and 11.3%, respectively, followed by the 8.6% and 10% as the second clusters in each. The results have shown the more diverse conformations for Islet(ε)-Aβ40(εεε) 1st and Islet(ε)-Aβ40(δδδ) 2nd seeds compared with more distinct structures in Islet(δ)-Aβ40(δδδ) 1st and Islet(δ)-Aβ40(δδδ) 1st seed dimers. In addition, by looking at the clusters, specifically the first cluster in all four cases shown in Figure 7, it is clear that in Aβ40 the helical structures shifted more to nonhelical as compare with the amylin that later could lead to further β-strand formation in Aβ40. In a previous study, it was also shown that amylin binding to the Aβ42 changed the conformation of the helix region in Aβ more than hIAPP [51].

### 2.4. Free Energy Landscape (FEL)

**Figure 8:**
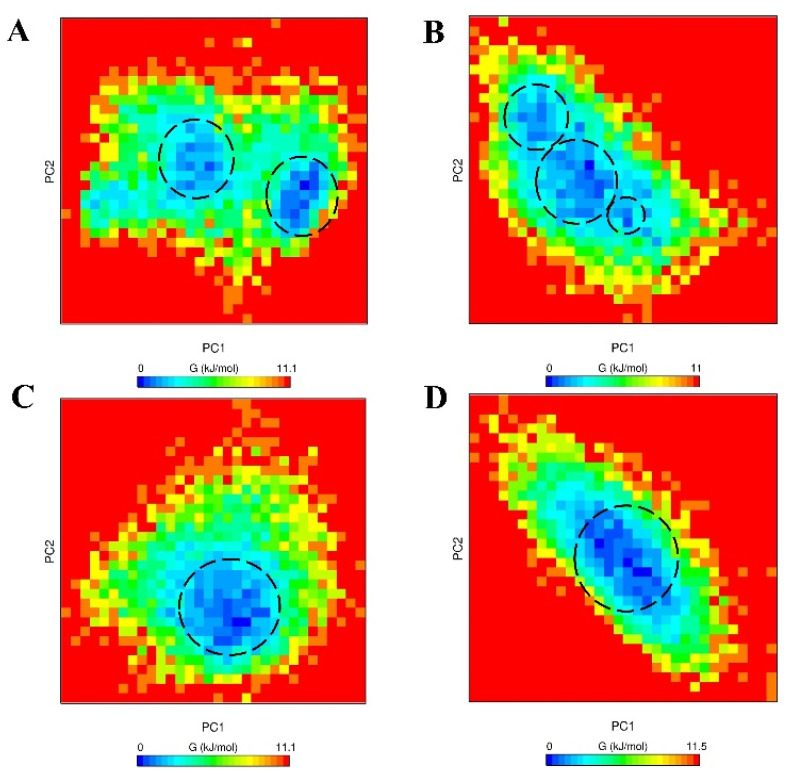
The Gibbs free energy landscape obtained during the last 50 ns MD simulation run as a function of first (PC1) and second (PC2) eigenvectors, respectively. (**A**) Islet(ε)-Aβ40(εεε) 1st, (**B**) Islet(ε)-Aβ40(δδδ) 2nd, (**C**) Islet(δ)- Aβ40(εεε) 2nd and (**D**) Islet(δ)-Aβ40(δδδ) 1st conformations. The minimal energy clusters are depicted in black dotted circles.

To clarify the conformational states of the dimers, the Gibbs free energy landscape was obtained using 2D projection of first (PC1) and second (PC2) eigenvectors. The principal component analysis (PCA) can determine the global motions of a protein within a few motions [57,58,59]. The color-coded FELs are represented in Figure 8. Analysis using the GROMACS v.5.0 software [56] exhibited the ΔG values vary between the range 0 to 11~11.5 kJ/mol for the chosen seeds of each dimer category. It can be determined that Islet(ε)- Aβ40(εεε) 1st and Islet(ε)-Aβ40(δδδ) 2nd may switch to assorted conformations as compared with other two structures due to more separated local minima and a higher energy barrier, which seems in agreement with the cluster analysis that implied the diverse conformations for these two dimers.

Meanwhile, Islet(δ)-Aβ40(εεε) 2nd and Islet(δ)-Aβ40(δδδ) 1st revealed less separated and more concentrated local minima clusters that could be in line with clustering analysis since the percentage of dominant clusters, in this case, were higher than Islet(ε)-Aβ40(εεε) 1st and Islet(ε)-Aβ40(δδδ) 2nd dimers as well.

### 2.5. Binding Free Energy

The binding free energy expressed by Mechanics-Poisson Boltzmann (MM-PBSA) method in GROMACS v.5.0 was used to calculate [60] the binding energies between two monomers in all seeds. The average molecular mechanics potential energy can be decomposed into Van der Waals (ΔE_vdw_), electrostatic (ΔE_elec_), and internal energies. The solvation free energy is also the summation of polar and non-polar solvation free energies. The combination of these two terms would result in the total binding free energies (ΔE_binding_) [60]. The calculated binding free energy terms have been shown in Table 1.

The values vary for each seed and all seeds showed the positive total binding free energies due to large polar solvation energy indicating that the structures in these forms still may not be completely stable. However, in some seeds, this value is smaller and closer to reach the stronger attraction. Meanwhile, in a previous study, the conformational energies for hexamers and dodecamers of Aβ42-amylin gave positive values for some conformations based on their orientations as well [45]. We should also consider the fact that usually reaching the well-folded structures requires at least ms simulation time which is computationally challenging [52]. In addition, some theoretical and experimental research has shown the Aβ and hIAPP pentamers as the more stable conformers and smallest oligomers as a seed for further aggregation [52]. It was also exhibited by an experimental study that at the initial stage of interaction in a mixture of Aβ and hIAPP the small oligomers were mostly unstable [16]. These could justify the reason behind the reduced stability of these structures at this level.

The decomposed terms in Table 1, showed that the electrostatic forces followed by the Van der Waals are the major contributors to the aggregation progress as it has been reported in pentameric aggregates as well [52]. However, the polar solvation free energy exhibited an unfavorable role during the aggregation of two chains. Whether this cross-interaction leads to faster and enhanced aggregation progress of Aβ and amylin or not is dependent on the ratio of the aggregates and strength of the connection between chains as well [52].

### 2.6. Hydrogen Bonding

Analysis of the formation of hydrogen bonds is of interest due to its pivotal role in protein motions and structural changes [61]. The average H-bonding between two chains during the last 50 ns of each trajectory for all seeds was calculated using the GROMACS and VMD software and shown in Table 2. As can be observed, except for the few seeds such as Islet(ε)-Aβ(δδδ) 2nd and Islet(δ)-Aβ(εεε) 3rd seeds, the others it seems did not show a significant difference.

Also, based on the donor-acceptor distance ~3 Å and angle cutoff set at 20 degrees [62], the H-bonding occupancy was calculated for the specified seeds. In the Islet(ε)-Aβ(εεε) 1st seed, the higher hydrogen bonds occupancies as compared with others were between THR4-ASN31 (54.2%), THR30-ILE26 (48.7%), HID13-TYR10 (31%), HIE6-HIE13 (29.8%), and HIE18-SER20 (19%). In the Islet(δ)-Aβ(εεε) 2nd seed, the hydrogen bonding occupancies between THR4-ASN31(54.2%), THR30-ILE26 (48.7%), HIE13- TYR10 (31%), HIE6-HIE13 (29.8%), HIE18-SER20 (19%) were higher than the other pairs. For the second seed of the Islet(ε)-Aβ(δδδ) conformer, the highest values were observed between THR30-ILE30 (57.7%), GLN10-THR36 (43.7%), HID14-GLU11 (22.5%), HID14-GLU11 (17.8%), HID13-HID14 (7.6%), and HID6-PHE4 (4.9%). In Islet(δ)-Aβ(δδδ), the highest occupancies were obtained between ASP7-VAL36 (62.2%), ALA8-LEU12 (52.3%), HID13-GLN15 (34.4%), HID14-GLN15 (33.4%), HID13-GLU11 (17%), HID14-VAL39 (15.5%), and HID18-SER19 (6.2%). From the results, we could see that histidine tautomers in the Aβ monomer contributed more to hydrogen bonding occupancy in the related chain compared with the histidine residue in the amylin chain.

## 3. Materials and Methods

The initial monomeric structures to make the dimers were taken from our previous works [20,31]. The crystal structure of Aβ40 and human amylin monomers had been taken from Protein Data Bank (PDB: 1BA4) and (PDB: 2KB8), respectively. The histidine residues were substituted with two N^ε^ –H or N^δ^–H tautomers. The 1:1 ratio of monomers was used to make the dimers. A total of four conformations were prepared for the simulation and three random seeds were simulated for each conformation. For each seed, the simulation was continued to reach the convergence and the last 50 ns of each trajectory was used for further analysis. The distance between the nearest atoms of two chains was set to ~5 Å for the initial structure. The chains were placed around 1.5 nm from the edge of the simulation box to avoid overlapping images. AMBER99SB force field implemented in GROMACS v.5.0 software (developed at the University of Groningen) [56] was applied to perform the MD simulations. The AMBER99SB force field has been widely and successfully used to study the aggregation progress of different proteins and peptides and has shown good agreement with experiments [63,64]. The structures were solvated using the TIP3P water model and neutralized using 1 Na^+^ ion before the MD run. Before the MD production, the structures are energy minimized using the steepest descent algorithm for a maximum of 50,000 steps with 0.01 minimization step size to relax the structure and avoid any steric clashes. After that, a 100-ps NVT equilibration has been conducted. The pressure of the system also has been equilibrated under an NPT ensemble. The simulations were done at 310 K (37 °C), which was controlled by the V-rescale method, and the particle-mesh Ewald (PME) method was applied for long-range electrostatic interactions [65]. The LINear Constraint Solver (LINCS) was used to constrain the lengths of the bonds [66]. MD simulations were performed using a 2 fs time step. The simulation length of Islet(ε)-Aβ40(εεε) seeds were 550, 450, and 590 ns. In the case of Islet(ε)-Aβ40(δδδ) dimer, seeds were simulated for 300, 300, and 350 ns. In the case of Islet(δ)-Aβ40(εεε) three seeds were simulated for 350, 420, and 250 ns. To get the convergence of Islet(δ)-Aβ40(δδδ) seeds, the simulation length was 250, 400, and 225 ns. Simulations, analysis, and visualizations were performed using the GROMACS v.5.0, visual molecular dynamics (VMD) v.1.9.2 (developed at the University of Illinois at Urbana-Champaign) [62], and UCSF Chimera v.1.12 (developed at the University of California) [67] software.

## 4. Conclusions

Herein, since the precise knowledge of cross-seeding between Aβ40 and amylin is still largely elusive, as the initial step, we attempted to present the cross-seeding between the Aβ40 and hIAPP monomers taking into account the histidine tautomerism effect. These models of cross-seeding in this study are just several of the many possible structural conformations that could be considered due to the complexity and diversified nature of amyloids. Herein, our results, through randomly seeded MD simulation, revealed the Aβ40(δδδ) isomer in cross-interaction with Islet(ε) and Islet(δ) isomers could maintain or enhance the β-sheet content in its structure that may make it more prone to further aggregation and have higher toxicity. The other isomers which did not have initial β-sheet content in monomeric forms did not show any generated β-sheet, except for one seed including Islet(ε) and Aβ40(εεε) that indicated a small amount of β-sheet. Our work represents the first step to initialize and investigate the histidine tautomerism effect on the cross-seeding of amylin and Aβ40 in dimeric form. So, studying the cross-talk of higher orders and more stable conformers of amyloids that were beyond the purpose of the current work is of interest for the near future. Discovering the feature changes of amyloidosis proteins during cross-interaction of Aβ40 and amylin considering all possible effective parameters such as tautomerism would be determined to design drugs that could help prevent toxic cross-seeding of amyloid peptides and developing AD in those with type 2 diabetes. It also would be helpful to find the possible cross-talk between T2D and AD at the atomic level.

## Figures and Tables

**Table 1:** Average values of binding free energy, Van der Waals (ΔE_vdw_), electrostatic (ΔE_elec_), polar solvation (ΔG_polar_), the solvent-accessible surface area (ΔG_sasa_) of all seeds. (Parenthesis indicate the standard error) (Energy units: kJ/mol).

Dimers	ΔE_vdw_	ΔE_elec_	ΔG_polar_	ΔG_sasa_	ΔE_binding_
Islet(ε)-Aβ(εεε) (1)	−151.013 (1.868)	−508.965 (5.505)	969.206 (10.696)	−19.514 (0.244)	289.111 (2.990)
Islet(ε)-Aβ(εεε) (2)	−300.080 (0.576)	−284.750 (0.705)	686.494 (1.496)	−35.159 (0.050)	66.520 (0.913)
Islet(ε)-Aβ(εεε) (3)	−348.255 (0.580)	−506.954 (0.983)	1021.628 (1.707)	−38.343 (0.051)	128.096 (1.065)
Islet(δ)-Aβ(εεε) (1)	−361.515 (0.752)	−449.112 (1.370)	1055.068 (2.821)	−40.605 (0.085)	203.830 (1.572)
Islet(δ)-Aβ(εεε) (2)	−302.575 (0.641)	−574.749 (1.589)	1249.936 (2.726)	−38.848 (0.059)	333.757 (1.397)
Islet(δ)-Aβ(εεε) (3)	−160.475 (0.343)	−269.774 (0.785)	596.982 (1.471)	−20.898 (0.041)	145.758 (0.951)
Islet(ε)-Aβ(δδδ) (1)	−274.357 (0.439)	−477.366 (0.575)	961.720 (1.081)	−29.931 (0.034)	180.096 (0.925)
Islet(ε)-Aβ(δδδ) (2)	−226.746 (0.532)	−243.161 (0.987)	577.932 (1.928)	−25.630 (0.051)	82.379 (1.186)
Islet(ε)-Aβ(δδδ) (3)	−274.619 (0.441)	−474.310 (0.677)	919.789 (1.201)	−28.932 (0.042)	141.888 (0.945)
Islet(δ)-Aβ(δδδ) (1)	−209.193 (0.407)	−224.150 (0.453)	503.763 (1.132)	−23.461 (0.039)	46.955 (0.858)
Islet(δ)-Aβ(δδδ) (2)	−258.789 (0.399)	−370.938 (0.879)	872.423 (1.706)	−26.770 (0.035)	215.987 (1.035)
Islet(δ)-Aβ(δδδ) (3)	−193.606 (0.467)	−247.322 (0.705)	572.019 (1.560)	−23.159 (0.048)	107.935 (1.245)

**Table 2:** The average hydrogen bonds formed between two chains during 50 ns of all seeds trajectories.

Dimers	Average H-Bond	Std
Islet(ε)-Aβ(εεε) (1)	6.8	1.9
Islet(ε)-Aβ(εεε) (2)	5.7	1.6
Islet(ε)-Aβ(εεε) (3)	6.2	1.3
Islet(δ)-Aβ(εεε) (1)	5.5	1.7
Islet(δ)-Aβ(εεε) (2)	4.8	1.6
Islet(δ)-Aβ(εεε) (3)	1.8	0.9
Islet(ε)-Aβ(δδδ) (1)	5.8	1.1
Islet(ε)-Aβ(δδδ) (2)	1.1	0.8
Islet(ε)-Aβ(δδδ) (3)	6.8	1.5
Islet(δ)-Aβ(δδδ) (1)	3.2	1.0
Islet(δ)-Aβ(δδδ) (2)	2.9	1
Islet(δ)-Aβ(δδδ) (3)	4.22	1.2

## Data Availability

The data presented in this study are available within the article, figures, tables.

## Abbreviations

Aβ	Aggregated amyloid β-peptide
AD	Alzheimer’s disease
APP	Amyloid protein precursor
hIAPP	Human islet amyloid polypeptide
MD	Molecular dynamics
RMSD	Root mean square deviation
SASA	Solvent accessible surface area
PCA	Principal component analysis
DSSP	Define Secondary Structure of Proteins
